# Supplementary material for: Clinicians’ communication with patients receiving a MCI diagnosis: The ABIDE project
Source: PLoS One. 2020 Jan 21;15(1):e0227282. doi: 10.1371/journal.pone.0227282 (PMC6974141; doi:10.1371/journal.pone.0227282)
Supplement: S1 Appendix — (DOCX) [file pone.0227282.s001.docx]

**Communicating individual (biomarker) test results**

- Are individual results of Neuropsychological assessment, Imaging, CSF by lumbar puncture, and/or Amyloid PET discussed/disclosed by the clinician?
- No
- Yes, communicated as being ‘normal’: e.g., results were within the normal range, no deviations, no impairments
- Yes, communicated as being ‘abnormal’: e.g. cognitive functions were impaired on one or more domains, a decrease in brain volume was shown, deviations from normal were shown in biomarker levels
- Yes, but unclear: results were discussed, but based on these results it is still unclear if cognitive functioning is abnormal or not, or the clinician’s communication about this test result is unclear.
- Does the clinician use (visual) aids to support the conversation?
- No aids are used
- Yes, MRI/CT images are used
- Yes, another type of aid is used, e.g., a drawing

**Diagnosis and cause**

- Is the term MCI or Mild Cognitive Impairment used by the clinician? (Yes / No)
- Are the following criteria addressed/discussed? (Yes / No)
  - Impairment in 1 or more cognitive domain?
  - No impairment with activities of daily living?
  - (emphasis on) No dementia/not demented?
- Is the (possible/potential) etiology/cause discussed/addressed?
  - Yes / No
  - What? E.g., (possible/potential) AD, Psychological problems/distress, Vascular damage? *Provide a quote.*
- Does the clinician explain the difference between AD and dementia? (Yes / No)

**Prognosis**

- Is the risk of developing dementia discussed by the clinician? (Yes / No)

Any type of dementia, or dementia in general. Words like risk, prognosis, chance, possibility can be used and the risk/chance has to be mentioned/described (see below ‘formats’ for examples of wording).

- Are the following risk communication formats used to discuss the risk of developing dementia? (Yes / No)
  - Verbal label: a verbal risk label is used, e.g., ‘the risk of developing dementia is small’, ‘there is a chance that you will develop dementia’ or ‘you could develop dementia’.
  - Percentages: the use of percentages to express the risk, e.g., ‘18% chance’
  - Natural frequencies: the use of natural frequencies, e.g., ‘1 out of 5’, or ‘10 out of 100’, etc.
  - Fifty-fifty: literally ’50-50’ chance or any equivalent, such as ‘half of patients will develop dementia, and the other half will not’.
  - Relative risk: the use of relative risk, e.g., ‘your chance is twice as high to develop dementia compared to the normal population’.
  - Reference class: a reference class is used when communicating the risk to develop dementia, e.g., ‘patients like you’ or ‘woman with your age’
  - Time frame: a time frame is used when communicating the risk to develop dementia, e.g., ‘in the next five years’ or ‘at some point in your life’
  - Negative and/or positive framing: does the clinician frame the likelihood of developing dementia (negative framing) and/or of not developing dementia (positive framing)?
- Is discussed that patient’s symptoms may decrease, stabilize, progress (increase)? (Yes / No)

**Next steps in (care) planning and symptom management**

- Are the following next steps addressed/discussed by the clinician? (Yes / No)
  - Further (additional) diagnostic testing (if Yes, then: what is the decision?)
  - Medication/medical treatment (*provide a quote*)
  - Follow-up consultations at the memory clinic
  - Life style adjustment / advice (*provide a quote)*
  - Study/trial participation
  - Driving ability testing
  - Follow-up by general practitioner (GP)
  - Referral to other care (e.g., psychologist, home care, physiotherapy, dietetics)
  - Other, e.g., genetic testing (*provide a quote)*
